# Hubs with Network Motifs Organize Modularity Dynamically in the Protein-Protein Interaction Network of Yeast

**DOI:** 10.1371/journal.pone.0001207

**Published:** 2007-11-21

**Authors:** Guangxu Jin, Shihua Zhang, Xiang-Sun Zhang, Luonan Chen

**Affiliations:** 1 Academy of Mathematics and Systems Science, Chinese Academy of Science, Beijing, China; 2 Graduate University of Chinese Academy of Sciences, Beijing, China; 3 Institute of Systems Biology, Shanghai University, Shanghai, China; 4 Department of Electrical Engineering and Electronics, Osaka Sangyo University, Osaka, Japan; 5 Exploratory Research for Advanced Technology (ERATO) Aihara Complexity Modelling Project, Japan Science and Technology Corporation (JST), Tokyo, Japan; 6 Institute of Industrial Science, The University of Tokyo, Tokyo, Japan; University of Nottingham, United Kingdom

## Abstract

**Background:**

It has been recognized that modular organization pervades biological complexity. Based on network analysis, ‘party hubs’ and ‘date hubs’ were proposed to understand the basic principle of module organization of biomolecular networks. However, recent study on hubs has suggested that there is no clear evidence for coexistence of ‘party hubs’ and ‘date hubs’. Thus, an open question has been raised as to whether or not ‘party hubs’ and ‘date hubs’ truly exist in yeast interactome.

**Methodology:**

In contrast to previous studies focusing on the partners of a hub or the individual proteins around the hub, our work aims to study the network motifs of a hub or interactions among individual proteins including the hub and its neighbors. Depending on the relationship between a hub's network motifs and protein complexes, we define two new types of hubs, ‘motif party hubs’ and ‘motif date hubs’, which have the same characteristics as the original ‘party hubs’ and ‘date hubs’ respectively. The network motifs of these two types of hubs display significantly different features in spatial distribution (or cellular localizations), co-expression in microarray data, controlling topological structure of network, and organizing modularity.

**Conclusion:**

By virtue of network motifs, we basically solved the open question about ‘party hubs’ and ‘date hubs’ which was raised by previous studies. Specifically, at the level of network motifs instead of individual proteins, we found two types of hubs, motif party hubs (mPHs) and motif date hubs (mDHs), whose network motifs display distinct characteristics on biological functions. In addition, in this paper we studied network motifs from a different viewpoint. That is, we show that a network motif should not be merely considered as an interaction pattern but be considered as an essential function unit in organizing modules of networks.

## Introduction

Many types of molecular networks display scale-free topologies which are characterized by the power-law degree distribution [1]–[5]. In spite of some negative remarks [6]–[9] on the studies of network structures, a small fraction of proteins generally interacting with many partners, i.e. so-called hubs, have attracted great interests [10]–[15] from the communities of both engineering and biology. To identify whether hubs vary their biological roles with the timing and location of the interactions, Han *et al.* proposed two types of hubs, i.e. ‘party hubs’ and ‘date hubs’, based on whether or not the hubs are co-expressed with their partners by using yeast microarray data [10]. The two distinct types of hubs not only display diverse spatial distribution for their partners but also organize the modules in different manners, where a module is referred as a group of physically or functionally linked molecules that work together to achieve a relatively distinct function [10], [16].

It should be noticed that the result of Han *et al*. on ‘party hub’ and ‘date hub’ was drawn from a filtered yeast interactome data (FYI). Recently, Batada *et al.* derived different results, in contrast to those of Han *et al*., based on another filtered yeast interactome data (*HC^fyi^*) manually curated from online publications [11] (see Materials and Methods). Due to the topological difference between FYI and *HC^fyi^*, Batada *et al.* found that there is no evidence for coexistence of party hubs and date hubs, and the results about ‘party hubs’ and ‘date hubs’ are totally not correct. Thus, the most striking question raised by them is whether or not the ‘party hubs’ and ‘date hubs’ truly exist in the networks.

In this paper, we aim to solve the contradiction between the two previous works. In virtue of network motifs, we define two new types of hubs in *HC^fyi^*, i.e. ‘motif party hub’ and ‘motif date hub’, which have the same characteristics as ‘party hub’ and ‘date hub’ respectively. Network motifs are the subgraphs that occur significantly more frequently in original network than random ones [17]–[22]. They have been revealed as the functional building blocks of biology networks [17], [18] and the spandrels of cellular complexity [23]. Similar to a previous research work on the role of network motifs in information processing [24], we focus on their important roles in acting as functional units in organizing modules. Moreover, in contrast to the previous studies on hubs, our work emphasizes on interactions of hubs and network motifs instead of individual proteins around hubs.

Specifically, we divide hubs into ‘motif party hubs’ (mPHs) and ‘motif date hubs’ (mDHs) based on the relationship between a hub's network motifs and protein complexes. In this paper, we demonstrate that the network motifs of an mPH (i.e. network motifs take the mPH as one of their nodes) are more likely to stay inside a protein complex with their mPH, control the local topological structure, locate in the same cellular localizations as the mPH, and co-express in microarray data. On the other hand, we reveal that the network motifs of an mDH tend to spread into different complexes, and act as the connectors among signal pathways, control the global topological structure, locate in different cellular localizations, and express differently in microarray data.

## Results

### Motif party hub (mPH) and motif date hub (mDH)

About 20% proteins, i.e. 197 proteins, in *HC^fyi^* were defined as hubs whose partners are not less than 12. There are 196 motif hubs with at least one network motif and only one hub without any network motif (see Methods and Materials and Table S1). Based on the relationship between a hub's network motifs and protein complexes, we divided the 196 motif hubs into 98 mPHs and 98 mDHs (see Methods and Materials). The quantitative criterion *Complex_ratio-same_* was defined to identify the relationship between a hub's network motifs and protein complexes. A relatively high *Complex_ratio-same_* implies that more network motifs of a hub (a network motif takes a hub as one of its nodes, and a hub may be used by multiple network motifs) belong to the same protein complex as the hub, e.g. the four proteins or three proteins in such a network motif are more likely to be in just one protein complex. Otherwise, it indicates that less network motifs of the hub belong to the same protein complex, e.g. the four proteins or three proteins in such a network motif are more likely to be parts of different protein complexes. From the definition of mPHs and mDHs, we can see that the network motifs of an mPH more likely stay together in the same protein complex as the mPH while those of an mDH spread outside the protein complex of the mDH.

**Figure 1:**
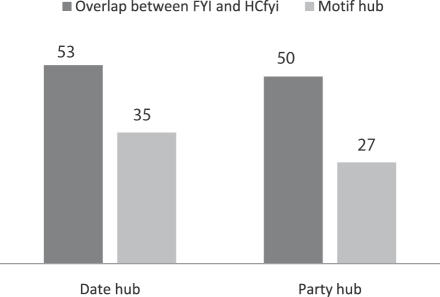
The hub overlap of FYI and *HC^fyi^* and the correctly divided mPHs and mDHs in the overlap.

In this paper, mPHs and mDHs defined by network motifs and protein complexes were introduced to study hubs at the level of network motifs instead of individual proteins (or nodes), so as to solve the open question whether or not *HC^fyi^* contains date hubs and party hubs. Due to the topological distinction between *HC^fyi^* and *FYI* (*HC^fyi^* looks like stratus while *FYI* looks like altocumulus [11]), 103 hubs were found in the overlap between 199 hubs in *FYI* and 197 hubs in *HC^fyi^*. We found that more than 60% of ‘party hubs’ and ‘date hubs’ defined by Han *et al.* have been correctly divided into mPHs and mDHs respectively by our method among the overlapped 103 hubs between FYI (proposed by Han *et al.*) and *HC^fyi^* (proposed by Batada *et al.*) (see Figure 1). Furthermore, we can see that the motifs of mPHs and mDHs have the same characteristics as the partners of party hubs and date hubs respectively in cellular localization or spatial distribution, controlling topological structure, linking with signal pathways and co-expression in microarray data. Therefore, we call them as motif ‘party’ and motif ‘date’ hubs due to the facts that they are similar to ‘party’ and ‘date’ hubs mentioned in Han *et al*. [10].

### Distinct cellular localizations for mPHs and mDHs

**Figure 2:**
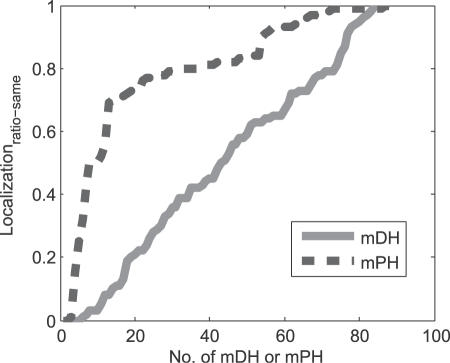
The spatial distribution of hubs.

One of main distinctions between party hubs and date hubs is their different spatial distributions (partners of date hubs are significantly more diverse in spatial distribution than those of party hubs) [10]. In virtue of another criterion, i.e. *Localization_ratio-same_* (see Materials and Methods), we found that *Localization_ratio-same_*s of mPHs are relatively high and those of mDHs are relatively low. The criterion *Localization_ratio-same_* shows localization relationship of network motifs of a hub. A higher one implies that three proteins or four proteins in each of network motifs of a hub are likely to locate in the same cellular localization as the hub. On the other hand, a lower one shows that the proteins in each of network motifs of a hub more likely locate in different cellular localizations. In Figure 2, mPHs have significantly higher *Localization_ratio-same_*s than mDHs (mean of mPHs: 0.7926; mean of mDHs: 0.4826; *P*<10^−11^ for Mann-Whitney U test.). Thus we can say that the network motifs of mPHs and mDHs have significantly different spatial distributions.

**Figure 3:**
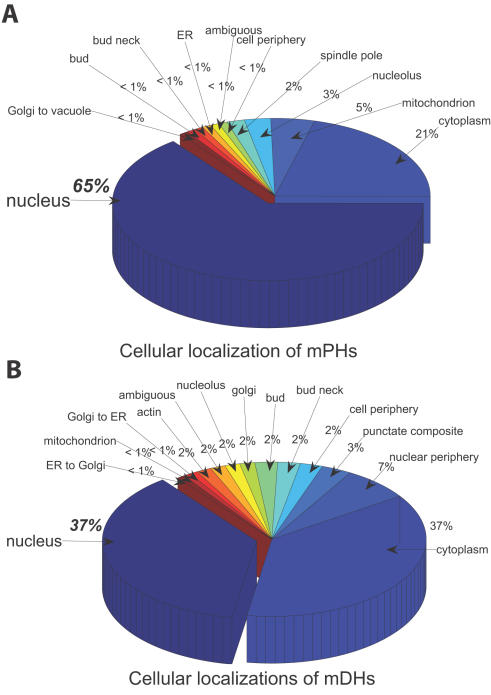
The localizations for mDHs and mPHs. (A) Cellular localizations of mDHs. (B) Cellular localizations of mPHs.

In our analysis, ‘nucleus’ and ‘cytoplasm’ were not excluded from the cellular localization data (see Materials and Methods). In this respect, our method is also different from one of Han *et al*. who excluded the ‘nucleus’ and ‘cytoplasm’ from the cellular localization data. Moreover, we found that mPHs and mDHs have a significant difference in these two cellular localizations. Most mPHs (about 65%) are located in the nucleus. However, most mDHs (about 63%) are localized in subcellular compartments other than the nucleus (see Figure 3). It is clear that there is a statistically significant localization difference between mPHs and mDHs (*x* ^2^ = 15.69, *P*≤0.001 for chi-square test). Therefore, mPHs prefer to ‘nucleus’ of a cell while mDHs are likely outside ‘nucleus’.

The cellular localization distribution of hubs and their network motifs implies that, mPHs with their network motifs tend to locate in nucleus while mDHs are more likely to locate outside nucleus, and their network motifs have a scattered spatial distribution.

### mPHs and mDHs control network architecture differently

**Figure 4:**
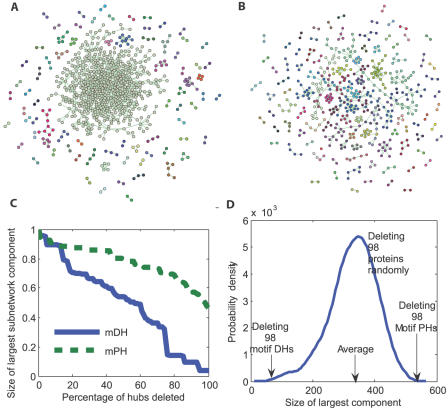
Deleting mPHs with their motifs and mDHs with their motifs respectively. (A) The case of deleting mPHs and their motifs. In this case, the main components remain (the points in the same color are in the same component). (B) The case of deleting mDHs and their network motifs. Deleting the mDHs and their motif proteins causes the main components to disappear (the points in the same color are in the same component). (C) Deleting hubs one by one. The *HC^fyi^* network is tolerant for the deletion of mPHs with their motifs. However, it is not tolerant for the deletion of mDHs with their motifs. (D) Deleting a randomly chosen set of 98 hubs with their motifs. We repeat the removal of 98 hubs with their motifs randomly for 1000 times. The sizes of the largest remaining components are all less than 555 that are the size of the largest component after removing the mPHs with their motifs. The sizes of the largest remaining components are all larger than 47 that are the size of the largest component after removing the mDHs with their motifs. Empirical P values are both less than 10^−3^. Biolayout [53] has been used to produce the figures in (A) and (B).

In early work, it has been shown that the *HC^fyi^* network is tolerant to hubs' deletion, which means that the key components of the *HC^fyi^* network still remain after removal of date hubs or party hubs, or even all the hubs [11]. One of direct reasons is that the protein-protein interactions in the network are too dense to be broken into fragments by only removing ‘date hubs’ or ‘party hubs’. Therefore, hubs rarely have effect on the structure of the network in such a case. However, hubs may affect the network topological structure in a different manner. In this paper, a new approach for breaking down both the hubs and their motifs from the network was introduced based on mPHs and mDHs. In Figure 4A, it appears that deleting the mPHs and their motifs has little influence on the main network structure, whereas deleting the mDHs and their motifs makes the network broken into many fragments in Figure 4B. In other words, mDHs and their motifs clearly have a global effect on the network structure. In addition, we also evaluated the p-values for the cases by removing mPHs with their motifs and by removing mDHs with their motifs in Figure 4D ( both of them are less than 0.001). Moreover, in Figure 4C, about 50% of proteins are still connected in the largest component after removal of mPHs (Figure 4A) and their network motifs while only less than 10% of proteins are connected in the largest component after removal of mDHs and their network motifs (Figure 4B). In Table 1, we can see that the largest component after deletion of mPHs and their network motifs (Figure 4A) contains 533 protein-protein interactions while it contains only 47 protein-protein interactions after deletion of mDHs and their network motifs (Figure 4B). Clearly, those results demonstrate that the mPHs mainly control the local structure by their motifs while the mDHs control the global structure by their motifs.

**Table 1:** Sizes and Numbers of the network components after removal of mPHs and mDHs respectively

Sizes and Numbers of subnetworks after removal of mPHs																		
Size	2	3	4	5	6	8	9	10	555									
Number	52	9	6	5	1	1	1	1	1									
Sizes and Numbers of subnetworks after removal of mDHs																		
Size	2	3	4	5	6	8	9	10	11	13	14	15	19	21	22	26	43	47
Number	48	14	8	8	4	1	2	2	1	1	1	1	1	1	1	1	1	1

### mPHs and mDHs link with signal pathways in different ways

**Figure 5:**
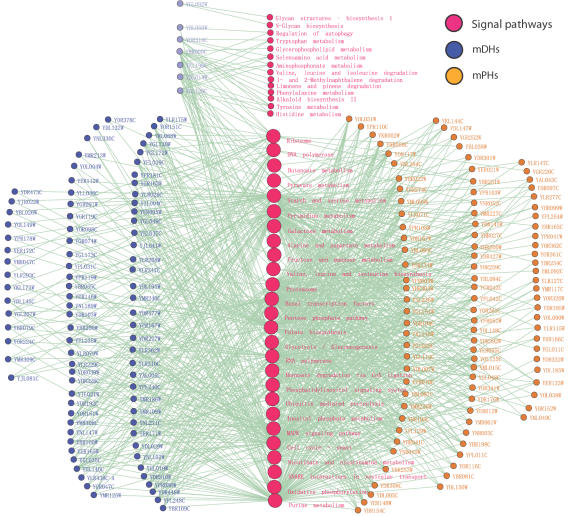
Network motifs linking up pathways with mDHs and mPHs. In the middle of the panel, the red circles are abstract representations of pathways, where 14 smaller ones only connect with 6 mDHs (circles in light blue), and 26 larger ones connect with not only mDHs in blue circles (except the light blue one at the top of the panel) but also mPHs in orange circles. If more than one motif proteins of an mDH or an mPH are involved in one pathway, we connect the mDH or mPH with the pathway by an edge. Biolayout [53] has been used to produce the figure.

The definitions of mPHs and mDHs imply that most network motifs of an mPH stay together with the mPH in a protein complex while the network motifs of an mDH are not restricted in a protein complex. Furthermore, we built up a network composed of hubs and signal pathways shown in Figure 5, by which we found that mDHs are more likely to be the connectors among signal pathways. If at least one of a hub's network motifs takes one or more proteins in some signal pathway as its node, there is one link between the hub and the signal pathway in the network as shown in Figure 5. It is not difficult to see that mDHs link with more signal pathways than mPHs (see Table 2, *x* ^2^ = 5.02, *P*<0.05 for Chi-square test). In addition, each mDH links with several signal pathways, i.e. the degrees of about 90% (86/98) of mDHs are all larger than 2, and their mean value is 4.3 (see Figure S1). As a result, mPHs and their network motifs mostly stay inside protein complexes, whereas mDHs and their motifs act as connectors among signal pathways.

**Table 2:** Linking with signal pathways by network motifs

	Linked pathways	Not linked pathways
mPHs	26	40
mDHs	40	26

There are totally 66 pathways in KEGG appearing in *HC^fyi^* network.

### Network motifs of mPHs are more co-expressed than those of mDHs

Another of main distinctions between party hubs and date hubs is whether or not the hubs are co-expressed (or are expressed simultaneously) with their partners [10]. Han *et al*. took the average PCC (or APCC) of the hubs as a measure to distinguish party hubs (with relatively high APCC) from date hubs (with relatively low APCC), where PCC is a Pearson correlation coefficient between a hub and one of its partners in microarray data. In this paper, however, we take another criterion to measure whether or not the network motifs of hubs are co-expressed. Specifically, we considered the standard deviation of average motif correlations (SAMC) of a hub (see Materials and Methods), in which average motif correlation (AMC) of a network motif is the average value of Pearson correlation coefficients between two proteins connected in the network motif. If SAMC is relatively low, the network motifs of the hub are more likely expressed at the same time (or say, the expression difference among the hub's network motifs is small). Moreover, besides the difference between APCC and SAMC in considering individual partners and network motifs respectively, it seems that SAMC is not equivalent to APCC while another one, i.e. mean of average motif correlations (MAMC), is similar to APCC. Such facts were actually confirmed by the numerical experiments. That is, by numerical experiments, we found that the MAMCs of mPHs have no significant difference from those of mDHs but the SAMCs of mPHs are significantly lower than those of mDHs (see Table 3). Therefore, in this paper, if there is no significantly difference in the average expressions of network motifs (MAMCs) between mPHs and mDHs, it is more confident for us to conclude that the network motifs of mPHs are more likely to be co-expressed than those of mDHs depending on the significant SAMC difference between mPHs and mDHs.

**Table 3:** Statistic significance for the differences of SAMCs between mDHs and mPHs in Microarray data

Mricroarray data	Point	SAMCs			MAMCs		
		Mean of mDHs'	Mean of mPHs'	P-value*	Mean of mDHs'	Mean of mPHs'	P-value*
Compendium	315	0.1380	0.1016	8.1349e-10	0.2481	0.2972	0.6207
Stress response	174	0.1512	0.1121	1.1546e-14	0.2569	0.2940	0.8580
Cell cycle	77	0.1233	0.1101	0.0065	0.1399	0.2080	0.0281
Pheromone treatment	45	0.1511	0.1394	0.0018	0.1100	0.1710	0.0386
Unfolded protein response	10	0.2461	0.2392	0.0386	0.1399	0.2080	0.0281
Sporulation	9	0.2391	0.2491	0.7628	0.2044	0.2814	0.2822

* Mann-Whitney U test [54].

## Discussion

At the level of network motifs instead of individual proteins, we found two types of hubs, motif party hubs (mPHs) and motif date hubs (mDHs), whose network motifs display distinct characteristics in organizing modules, cellular localizations, controlling network architecture, and co-expression in microarray data. More importantly, such a result answered the open question whether or not *HC^fyi^* contains ‘date hubs’ and ‘party hubs’, i.e. the contradiction on ‘date hubs’ and ‘party hubs’ between works of Han *et al*. [10] and Batada *et al*. [11], at the level of network motifs. Moreover, more results on degree and cluster coefficient differences (see Figure S2) and GO function difference between mPHs and mDHs (see Figure S3) were also found (see Text S1).

Although our results support the observation on ‘party hub’ and ‘date hub’ conducted by Han *et al*. [10], our analysis methods are totally different from theirs. That is, their study focus remains at the level of individual proteins (or nodes), however, our work is at the level of network motifs. The main procedures of the proposed method based on network motifs can be summarized as follows:

1. First, dividing hubs by considering how many network motifs stay together with their hubs in protein complexes;
2. Second, constructing spatial distribution of hubs by considering how many hubs' network motifs locate in the same cellular localizations as the hubs;
3. Third, controlling the network architecture of hubs by considering the important roles of network motifs in breaking down network topological structure;
4. Fourth, organizing the modules by considering the roles of network motifs in linking hubs and signal pathways;
5. Lastly, analyzing co-expression in microarray data of hubs by considering the difference among network motifs' expressions.

**Figure 6:**
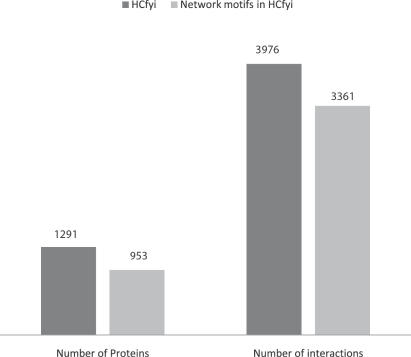
The proteins and interactions in *HC^fyi^* and in all network motifs of *HC^fyi^* respectively.

Why were network motifs adopted to distinguish ‘party hubs’ from ‘date hubs’ in *HC^fyi^* in this work? One of main reasons is that we surprisingly found that all network motifs in *HC^fyi^* occupy about 74% proteins and involve 85% interactions of *HC^fyi^* (see Figure 6). In other words, from the viewpoint of network motifs, the main structure of *HC^fyi^* is composed of network motifs rather than individual proteins. Thus, it is natural that we adopt network motifs as main elements while studying hubs. Another reason is that the appropriate size of the chosen network motifs, i.e. 3 or 4, determines their important role in characterizing both small size elements, i.e. molecules, at the ‘low level’ of a network, and large size elements, i.e. modules such as protein complexes [25]–[28] and signal pathways [29], [30], at the ‘high level’ of a network. In our analysis, we did study the network from different levels [31]. For example, in our analysis, mPHs' network motifs control a local topological structures and stay together inside protein complexes, which represents a ‘lower level’ of the network. On the other hand, mDHs' network motifs control the global topological structure and act as the connectors among signal pathways, which represent a ‘high level’ of the network. At either ‘low level’ or ‘high level’ of the network, the network motif is a suitable and essential building block or functional unit to characterize both relatively small elements, i.e. molecules, at a ‘low level’, and relatively large ones, i.e. modules, at a ‘high level’.

We studied the biological network of yeast from the viewpoint of network motifs in the paper, in particular stressing on their biological roles in biological networks rather than the topological structures of network motifs. Both theoretical and numerical analysis show that network motifs should not be merely considered as a connection pattern from topological structures but be considered as essential function units in organizing the modules from biological processes[24].

## Materials and Methods

### Protein interaction data

The FYI dataset of 2491 interactions among 1375 proteins was obtained from Han *et al*. [10]. The *HC^fyi^* dataset of 3976 interactions among 1291 proteins was obtained from Batada *et al*. [11]. The methodologies to construct FYI and *HC^fyi^* are similar. They were both based on an intersection method in which only the interactions observed at least twice are retained from various datasets. Datasets of the FYI were derived from HTP [25], [26], [32]–[34], APT [34]–[36], in silico-predicted dataset [37]–[39] and MIPS [40]. Datasets of the *HC^fyi^* were derived from all extent protein interaction datasets, which include all LC interaction data (BioGRID [41], BIND [36], DIP [42], MINT [43], and MIPS [40]), and all HTP interaction data [25]–[28], [34], [35], [42]. Especially, the LC data were manually curated from over 31,793 abstracts and online publications [41], and there is no interaction derived from standard large-scale experiments in both FYI and *HC^fyi^*.

### Protein complex, signal pathway and cellular localization data

The protein complex data were derived from MIPS [40] in September of 2006 and the signal pathway data were derived from KEGG [44] in November of 2006. Cellular localization data were derived from Huh *et al*. [45].

### Gene expression data

The 6 microarray datasets [10], [11], [46] (Stress response [47], cell cycle [48], pheromone treatment [49], unfolded protein response [50], sporulation [51] and compendium [46]) were normalized with Z score normalization [52] (i.e. the expression measurement for each gene was adjusted to have a mean of 0 and a standard deviation of 1) using the original log_2_ fold change values. Compendium gene expression data are an expression-profiling compendium of 315 data points for most yeast genes across other five different experimental conditions. The PCC (Pearson correlation coefficient) of motifs were calculated for the five conditions and the combined set of all conditions (compendium).

### Hub

We selected about top 20% proteins with relatively more partners, i.e. 197 proteins, in the *HC^fyi^* network, which are defined as hubs. All of their partners are not less than 12. There are 103 hubs in the overlap of hubs of *HC^fyi^* and FYI (199 hubs).

### Network Motifs detected by mfinder1.2

In consideration that the protein-protein interaction networks are undirected, the network motifs appearing in these undirected networks are undoubtedly undirected. For three-node substructures, only one network motif, i.e. triangle or ID: 238, has been found, whose Z-score is 317.43. For four-node substructures, one network motif, i.e. square or ID: 13260, has been found and been chosen as the representative four-node network motif in our study, whose Z-score is 12.90. Indeed, for four-node network motifs, others have also been found. The reason why we chose the square four-node network motif is that the other four-node network motifs can be composed of the triangle and the square. The network motifs were found by mfinder1.2 [17], [18].

### Division of hubs into mPHs and mDHs

We propose a quantitative criterion to divide hubs into mPHs and mDHs in this paper. According to the quantitative criterion *Complex_ratio-same_*, we can have a partition of hubs based on the relationship between a hub's network motifs and protein complexes. If a protein *H* is a hub in FYI, and *M* is a set of the hub's network motifs that are composed of *M*_1_, *M*_2_,…, *M*_|*M*|_ (for every network motif *M_i_*, *i*∈{1,2,…,|*M*|}, *H*∈*M_i_* must be satisfied) , then we have
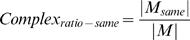
where set *M_same_* is composed of those network motifs whose three proteins or four proteins all belong to just the same protein complex. In other words, for the protein *P_j_* in some network motif *M_k_*, *j*∈{1,2,…,|*M_k_*|}, the protein complex set of *P_j_* is *Complex_j_* that is composed of those protein complexes containing the protein *P_j_*. If 
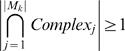
, then *M_k_*∈*M_same_*. |•| is the number of elements in some set. Thus, 98 hubs with relatively high *Complex_ratio-same_* (larger than or equal to 0.5) are called mPHs, and 98 hubs with relatively low *Complex_ratio-same_* (less than 0.5) are called mDHs (see Table S1).

### A measure for cellular localizations

According to the quantitative criterion *Localization_ratio-same_* proposed in this paper, we can measure the spatial distribution of a hub's network motifs. If a protein *H* is a hub in FYI, and *M* is a set of the hub's network motifs that are composed of *M*_1_, *M*_2_,…, *M*_|*M*|_, then we have
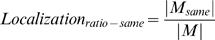

where set *M_same_* is composed of those network motifs whose three proteins or four proteins all locate in just the same cellular localization. In other words, for the protein *P_j_* in some network motif *M_k_*, *j*∈{1,2,…,|*M_k_*|}, the cellular localization set of *P_j_* is *Localization_j_* that is composed of those cellular localizations of the protein *P_j_*. If 
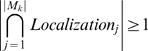
 , then *M_k_*∈*M_same_*. |•| is the number of elements in some set.

### Standard deviation of Average Motif Correlation (SAMC)

To analyze co-expression or study the difference in gene expression of a hub's network motifs, we define Standard deviation of Average Motif Correlation (SAMC). Average Motif Correlation (AMC) for one of a hub's network motifs is to measure the average gene expression level for the network motif. If a protein *H* is a hub in FYI, and *M* is a set of the hub's network motifs that are composed of *M*_1_, *M*_2_,…, *M*_|*M*|_. For each network motif *M_i_* (*i*∈{1,2,…,|*M*|}) that contains three proteins or four proteins as mentioned in the section about network motifs, then we have
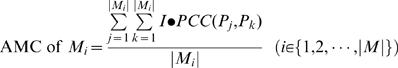

where |*M_i_*| is the number of interactions in network motif *M_i_*, i.e. 3 or 4, and *P_j_*, *P_k_* are any two proteins in *M_i_*. *PCC*(*P_j_*, *P_k_*) is the Pearson correlation coefficient between proteins *P_j_* and *P_k_*. *I* is a function defined as equal to 1 if *P_j_*, *P_k_* are linked in the network motif *M_i_*, and equal to 0 if *P_j_*, *P_k_* are not linked in the network motif *M_i_*.

Thus, SAMC is the Standard deviation of all AMCs for all network motifs of a hub.

### Mean of Average Motif Correlation (MAMC)

Mean of Average Motif Correlation (MAMC) is the Mean of all AMCs for all network motifs of a hub.

## Supporting Information

Text S1 Additional results on mPHs and mDHs.
(0.11 MB PDF)

Table S1 mPHs and mDHs.
(0.02 MB PDF)

Figure S1 The degrees of mDHs in Figure 5.
(0.08 MB TIF)

Figure S2 The degree and cluster coefficient differences between mPHs and mDHs.
(0.07 MB TIF)

Figure S3 Function_ratio-sames of mDHs is significantly different from those of mPHs
(0.04 MB TIF)
