# Supplementary material for: Hubs with Network Motifs Organize Modularity Dynamically in the Protein-Protein Interaction Network of Yeast
Source: PLoS One. 2007 Nov 21;2(11):e1207. doi: 10.1371/journal.pone.0001207 (PMC2065901; doi:10.1371/journal.pone.0001207)
Supplement: Text S1 — Additional results on mPHs and mDHs. (0.11 MB PDF) [file pone.0001207.s001.pdf]

TextS1 for 'Hubs with network motifs organize modularity dynamically in a protein interaction network of yeast'

Guangxu Jin[1,2] Shihua Zhang[1,2] Xiang-Sun Zhang[1*] Luonan Chen[3,4,5*]

1 Sparse connection of motifs for mDHs and dense connection of motifs for mPHs

In the HC^{fyi} network, an mDH or an mPH almost has the same number of partners (Mean of mDH's degree is 16.99 and Mean of mPH's degree is 17.64. $p = 0.4922$ for student t-test) (see Figure S2A). This is in contrast to the result in FYI of Han et al. that a date hub has more partners than a party hub. Moreover, the network motifs of mPHs are more significantly dense than those of mDHs (Mean of mDH's cluster coefficients is 0.1654 and Mean of mPH's cluster coefficients is 0.1654. $p < 10^{-6}$ for student t-test) (see Figure S2B). Thus, the motifs of mDHs are connected sparsely and the motifs of mPHs are connected densely.

2 The motifs of an mPH have higher probability performing the same function than those of an mDH.

$\text{Function}_{ratio-same}$s of mDHs and mPHs are shown in Figure S3. Clearly, $\text{Function}_{ratio-same}$s of mDHs are significantly different from ones of mPHs (mean of mDHs: 0.1722, mean of mPHs: 0.4500, $p < 10^{-8}$ for Mann-Whitney U test). Furthermore, the different localization preferences for the motifs of mPHs and mDHs may explain this result. The reason why the motifs of mPHs have similar functions is that mPHs with their motifs are likely to be inside 'nucleus' and mainly perform the functions within the 'nucleus', which allows the motifs of mPHs to have the same functions. In contrast, the motifs of mDHs are likely to be located outside the 'nucleus' and perform different tasks, thereby functioning in different ways.

As shown in above, by network motifs, mPHs and mDHs not only display their different roles in controlling the network topological structure and characterizing the functions of modules, but also show their different natures in co-expression and co-function. In the following, we will make further analysis about the important roles of mPHs and mDHs in controlling the network topological structure as well as modules.

3 About 90% mDHs link with more than 2 pathways.

In Figure S1, we can easily find that about 90% can link with more than 2 signal pathways and the mean of mDHs' degrees in Figure 5 is 4.3 (see Figure S1).

*corresponding author: zxs@amt.ac.cn, chen@eic.osaka-sandai.ac.jp

4 The network motifs of mDHs and mPHs are essential to the functions of signal or metabolic pathways.

In Figure 5, for convenience, we call the center 26 pathways as ‘DP-pathways’ that involve the motif proteins of both mDHs and mPHs, the above 14 pathways as ‘D-pathways’ that only involve the motif proteins of mDHs, and the other 26 pathways in Table 2 as ‘B-pathways’ that do not involve any motif protein of mDHs or mPHs.

4.1 mPHs with their motifs are the weatherglass to the functions of signal or metabolic pathways.

As shown in Figure 2, the motifs of an mPH prefer to ‘nucleus’ and tend to stay together with the mPH. Therefore, not surprisingly, the motifs of mPHs are only connected to DP-pathways, and are related to less than half of the pathways. Interestingly, in Table S3, the number of mPHs, at least one of whose motif proteins are involved in some DP-pathway, can represent the extent of the correlation between the pathway and ‘nucleus’.

DP-pathways with a large number of mPHs are mainly responsible for the biochemical reactions in ‘nucleus’. In the first part of Table S3, it is clear that the number of mPHs involved in these DP-pathways is high. A striking feature of the pathways is that most of them are critical to DNA, RNA and the transcription process in ‘nucleus’. First, ‘Purine’ and ‘Pyrimidine’ metabolism are needed to make DNA and RNA, and ‘Folate biosynthesis’ is to synthesize DNA and RNA. Second, ‘Basal transcription factors’ and ‘RNA polymerase’ are responsible for the transcription of DNA. Third, ‘Proteasome’ can degrade the proteins in ‘nucleus’. In addition, ‘Cell cycle’ is to divide the cell, including the division of ‘nucleus’. Therefore, the important feature of these DP-pathways with a large number of mPHs is that their biochemical reactions are mainly related to the ‘nucleus’.

DP-pathways with a small number of mPHs are mainly responsible for the biochemical reactions in ‘organelle’ and ‘cytoplasm’. As shown in the second part of Table S3, the number of mPHs involved in these DP-pathways is low. A striking feature of these DP-pathway is that their biochemical activities are related to the translation of proteins, folding, sorting and degradation of proteins, as well as energy and carbohydrate metabolism, which mainly occur in the ‘organelle’ and ‘cytoplasm’ of a cell. First, ‘Ribosome’, ‘Alanine and aspartate metabolism’, ‘Valine, leucine and isoleucine biosynthesis’ are needed to translate mRNAs to proteins. Moreover, ‘Ubiquitin mediated proteolysis’ and ‘SNARE interactions in vesicular transport’ degrade or fold the proteins. Second, ‘Oxidative phosphorylation’ is the ‘Energy Metabolism’, which occurs in the ‘mitochondria’, and ‘MAPK signaling pathway’ is a ‘Signal Transduction’, in which the initiating event occurs at the cell surface, but the final event occurs in the nucleus. Third, the others mainly contribute to the ‘Carbohydrate Metabolism’, such as that ‘Glycolysis / Gluconeogenesis’, ‘Pyruvate metabolism’, ‘Butanoate metabolism’, ‘Pentose phosphate pathway’, ‘Fructose and mannose metabolism’ and ‘Galactose metabolism’. Therefore, the DP-pathways perform their biochemical role in ‘organelle’ and ‘cytoplasm’.

D-pathways and B-pathways with no mPH are mainly responsible for the biochemical reactions between the surface of a cell or membrane and ‘cytoplasm’. There are 40 pathways with no mPH in the third part of Table S3 and in Table S2, which include ‘Amino Acids Metabolism’ (13 pathways), ‘Metabolism of Other Amino Acids’ (2 pathways), ‘Lipid Metabolism’ (5 pathways), ‘Glycan Biosynthesis and Metabolism’ (6 pathways), ‘Energy Metabolism’ (2 pathways) , ‘Carbohydrate Metabolism’ (3 pathways) and others (see Table S4). It should be noted that the lipid is the main component of membrane proteins, such as that ‘Fatty acid metabolism’ in ‘Lipid Metabolism’ takes the fatty acids from the extracellular to intracellular through the ‘membrane’. Therefore, the pathways contribute not only to the metabolism of amino acids but also to the transducing and storing energy, which mainly come from the outside of the cell.

As a result, the presence of mPHs is an important signal which can indicate the functions of

pathways. More mPHs a pathway includes, more larger the extent of the correlation is between the pathway and 'nucleus'. Therefore, the mPHs can be viewed as a weatherglass, which tells us whether a pathway is related to 'nucleus' in a cell.

4.2 DP-pathways in 'nucleus' are more likely related to mPHs' motif proteins while DP-pathways outside 'nucleus' prefer to mDHs' motif proteins.

Interestingly, in Table S3, the contribution of mPHs to DP-pathways in 'nucleus' is larger than that of mDHs (mean of mPHs: 41.6667, mean of mDHs: 21.8333, $P < 0.01$ for Wilcoxon rank sum test), whereas the contribution of mPHs to DP-pathways outside 'nucleus' is smaller than that of mDHs (mean of mPHs: 2.3571, mean of mDHs: 6.7857, $P < 0.01$ for Wilcoxon rank sum test). Now, we can conclude that the DP-pathways in 'nucleus' and 'cytoplasm' mainly involve the motifs of mPHs and mDHs respectively. The different tasks of the pathways in 'nucleus' and 'cytoplasm' can explain this interesting result. Since the elementary characteristic of 'nucleus' is to store the most important information of heredity in DNA, as well as facilitating transcription of DNA, thereby the pathways or metabolism related to 'nucleus' mainly accomplish the transcription of DNA within 'nucleus'. But the pathways outside 'nucleus' not only facilitate the tasks to obtain the code for protein translation, such as mRNA, but also need amino acids and energies from the outer world of the cell. Therefore, the pathways in 'nucleus' that perform the constrained task, i.e. transcription of DNA and preparation for protein translation inside the 'nucleus', involve more mPHs, whose motifs tend to stay together with mPHs, while the pathways outside 'nucleus' that need to reach a relatively large world to accomplish different tasks across the cell involve more mDHs, whose motifs tend to spread to other localizations of the cell.

In Table S3, an important feature of DP-pathways is that some of them related to 'nucleus' have motif proteins for most mPHs and mDHs, such as 'Basal transcription factors', 'Purine metabolism', 'Pyrimidine metabolism', 'RNA polymerase', 'Cell cycle', 'Folate biosynthesis', 'Ribosome' and 'DNA polymerase'. The above 8 DP-pathways include motif proteins for 70 mDHs and 86 mDHs. In contrast, the other 18 DP-pathways and 14 D-pathways in Table S3 all together include motif proteins for 61 mDHs and 76 mDHs. The result implies that most mDHs and mPHs are essential to the pathways related to 'nucleus'.

As a result, the number of mPHs whose motifs are involved in a pathway is a weatherglass which can tell whether the biochemical reaction of the pathway is related to 'nucleus'. Moreover, the pathways in 'nucleus' involve more mPHs' motif proteins whereas the pathways outside 'nucleus' involve more mDHs' motif proteins. Furthermore, many motif proteins for most mPHs and mDHs are favorite of some of DP-pathways in 'nucleus'.

Table S2: The linkages of the 26 pathways that motif proteins of mDHs and mPHs are not involved in

No. of pathways	pathways	proteins connected to motifs of mDHs	proteins connected to motifs of mPHs
00071	Fatty acid metabolism	YOL086C	
00120	Bile acid biosynthesis	YOL086C	
00561	Glycerolipid metabolism	YOL086C	
00980	Metabolism of xenobiotics by cytochrome P450	YOL086C	
00061	Fatty acid	YNR016C	
00710	Carbon fixation	YAL038W	
03060	Protein export	YDL092W YDR292C YPL210C YPL243W YPR088C	
00513	High-mannose type N-glycan biosynthesis	YEL036C	
00251	Glutamate metabolism	YJR109C YGL245W	
00860	Porphyrin and chlorophyll metabolism	YGL245W	
00970	Aminoacyl-tRNA biosynthesis biosynthesis	YGL245W YGR264C	
00640	Propanoate metabolism	YNR016C	
02021	Two-component system - Organism-specific	YLR006C	
00020	Citrate cycle (TCA cycle)	YFL018C YIL125W	
00260	Glycine, serine and threonine metabolism	YFL018C YER052C	YER052C
00310	Lysine degradation	YIL125W	
00300	Lysine biosynthesis	YER052C	YER052C
00271	Methionine metabolism	YGR264C	
00220	Urea cycle and metabolism of amino groups	YBR208C	YBR208C
00910	Nitrogen metabolism	YPR145W	YPR145W
00770	Pantothenate and CoA biosynthesis		
00563	Glycosylphosphatidylinositol(GPI)-anchor biosynthesis		
01031	Glycan structures - biosynthesis 2		
00400	Phenylalanine, tyrosine and tryptophan biosynthesis		
00330	Arginine and proline metabolism		
00530	Aminosugars metabolism		

No. of Pathways and names of Pathways are obtained from KEGG database [?].

Table S3: DP-pathways and D-pathways

No. of pathways	pathways	NDH	NPH
03022	Basal transcription factors	25	67
00230	Purine metabolism	35	58
00240	Pyrimidine metabolism	32	58
03020	RNA polymerase	22	56
04111	Cell cycle - yeast	36	51
03050	Proteasome	11	43
00500	Starch and sucrose metabolism	33	39
00790	Folate biosynthesis	30	36
04070	Phosphatidylinositol signaling system	13	26
00562	Inositol phosphate metabolism	8	22
00632	Benzoate degradation via CoA ligation	9	22
00760	Nicotinate and nicotinamide metabolism	8	22
03010	Ribosome	15	4
00010	Glycolysis / Gluconeogenesis	7	4
04120	Ubiquitin mediated proteolysis	6	3
00252	Alanine and aspartate metabolism	4	3
00290	Valine, leucine and isoleucine biosynthesis	4	3
00620	Pyruvate metabolism	4	3
00650	Butanoate metabolism	4	3
04010	MAPK signaling pathway	21	2
03030	DNA polymerase	15	2
00190	Oxidative phosphorylation	1	2
00030	Pentose phosphate pathway	2	1
00051	Fructose and mannose metabolism	2	1
00052	Galactose metabolism	2	1
04130	SNARE interactions in vesicular transport	8	1
00280	Valine, leucine and isoleucine degradation	1	0
00350	Tyrosine metabolism	4	0
00360	Phenylalanine metabolism	1	0
00564	Glycerophospholipid metabolism	1	0
00624	1- and 2-Methylnaphthalene degradation	1	0
00903	Limonene and pinene degradation	1	0
00960	Alkaloid biosynthesis II	1	0
04140	Regulation of autophagy	2	0
00340	Histidine metabolism	3	0
00380	Tryptophan metabolism	3	0
00440	Aminophosphonate metabolism	3	0
00450	Selenoamino acid metabolism	3	0
00510	N-Glycan biosynthesis	1	0
01030	Glycan structures - biosynthesis 1	1	0

No. of Pathways and names of Pathways are obtained from KEGG database [?]. NDH: the number of mDHs whose motifs are involved in. NPH: the number of mPHs whose motifs are involved in.

Table S4: Pathway catalogs of D-pathways and B-pathways		
No. of pathways	Pathways	Pathway catalogs
00280	Valine, leucine and isoleucine degradation	Amino Acid Metabolism
00350	Tyrosine metabolism	Amino Acid Metabolism
00360	Phenylalanine metabolism	Amino Acid Metabolism
00564	Glycerophospholipid metabolism	Lipid Metabolism
00624	1- and 2-Methylnaphthalene degradation	Xenobiotics Biodegradation and Metabolism
00903	Limonene and pinene degradation	Biosynthesis of Secondary Metabolites
00960	Alkaloid biosynthesis II	Biosynthesis of Secondary Metabolites
04140	Regulation of autophagy	Folding, Sorting and Degradation
00340	Histidine metabolism	Amino Acid Metabolism
00380	Tryptophan metabolism	Amino Acid Metabolism
00440	Aminophosphonate metabolism	Metabolism of Other Amino Acids
00450	Selenoamino acid metabolism	Metabolism of Other Amino Acids
00510	N-Glycan biosynthesis	Glycan Biosynthesis and Metabolism
01030	Glycan structures - biosynthesis 1	Glycan Biosynthesis and Metabolism
00071	Fatty acid metabolism	Lipid Metabolism
00120	Bile acid biosynthesis	Lipid Metabolism
00561	Glycerolipid metabolism	Lipid Metabolism
00980	Metabolism of xenobiotics by cytochrome P450	Xenobiotics Biodegradation and Metabolism
00061	Fatty acid	Lipid Metabolism
00710	Carbon fixation	Energy Metabolism
03060	Protein export	Folding, Sorting and Degradation
00513	High-mannose type N-glycan biosynthesis	Glycan Biosynthesis and Metabolism
00251	Glutamate metabolism	Amino Acid Metabolism
00860	Porphyrin and chlorophyll metabolism	Metabolism of Cofactors and Vitamins
00970	Aminoacyl-tRNA biosynthesis biosynthesis	Translation
00640	Propanoate metabolism	Carbohydrate Metabolism
02021	Two-component system - Organism-specific	Signal Transduction
00020	Citrate cycle (TCA cycle)	Carbohydrate Metabolism
00260	Glycine, serine and threonine metabolism	Amino Acid Metabolism
00310	Lysine degradation	Amino Acid Metabolism
00300	Lysine biosynthesis	Amino Acid Metabolism
00271	Methionine metabolism	Amino Acid Metabolism
00220	Urea cycle and metabolism of amino groups	Amino Acid Metabolism
00910	Nitrogen metabolism	Energy Metabolism
00770	Pantothenate and CoA biosynthesis	Glycan Biosynthesis and Metabolism
00563	Glycosylphosphatidylinositol(GPI)-anchor biosynthesis	Glycan Biosynthesis and Metabolism
01031	Glycan structures - biosynthesis 2	Glycan Biosynthesis and Metabolism
00400	Phenylalanine, tyrosine and tryptophan biosynthesis	Amino Acid Metabolism
00330	Arginine and proline metabolism	Amino Acid Metabolism
00530	Aminosugars metabolism	Carbohydrate Metabolism

No. of Pathways and names of Pathways are obtained from KEGG database.

References

[1] Wuchty S, Oltvai ZN, Barabsi AL (2003) Evolutionary conservation of motif constituents in the yeast protein interaction network. Nat Genet. 35(2):176-9

[2] Watts DJ, Strogatz SH (1998) Collective dynamics of 'small-world' networks. Nature. 393(6684):440-2.

[3] Vazquez A, Dobrin R, Sergi D, Eckmann JP, Oltvai ZN, et al. (2004) The topological relationship between the large-scale attributes and local interaction patterns of complex networks. Proc Natl Acad Sci U S A.101(52):17940-5.

[4] Dobrin R, Beg QK, Barabasi AL, Oltvai ZN (2004)Aggregation of topological motifs in the Escherichia coli transcriptional regulatory network. BMC Bioinformatics. 30;5:10. 1. Harris MA,

[5] Clark J, Ireland A, Lomax J, Ashburner M, et al. (2004) The Gene Ontology (GO) database and informatics resource. Nucleic Acids Res 32: D258-261.
