# Supplementary material for: Hubs with Network Motifs Organize Modularity Dynamically in the Protein-Protein Interaction Network of Yeast
Source: PLoS One. 2007 Nov 21;2(11):e1207. doi: 10.1371/journal.pone.0001207 (PMC2065901; doi:10.1371/journal.pone.0001207)
Supplement: Table S1 — mPHs and mDHs. (0.02 MB PDF) [file pone.0001207.s002.pdf]

Table S1

mDHs:	mDH	Degree	Motif density	Complex_ratio-same	Localization_ratio-same	Function_ratio-same
	CDC28	71	4.423	0.04	0.650	
	RPO21	40	21.225	0.26	0.940	0.19
	ACT1	33	3.424	0.08		0.00
	NUP116	30	27.667	0.01	0.030	0.17
	BRR2	28	42.214	0.10	0.770	0.66
	PSE1	27	13.185	0.15	0.280	0.00
	RAD23	26	32.846	0.13	0.790	0.00
	SMT3	24	1.708	0.00	0.420	0.00
	NUP100	24	25.583	0.01	0.130	0.14
	HHF1	23	3.174	0.23	0.420	0.01
	PRP31	23	29.609	0.42	0.730	0.70
	KAP95	23	20.000	0.02	0.220	0.00
	SNU114	23	41.391	0.44	0.720	0.68
	RPB3	22	29.682	0.21	0.790	0.30
	CMD1	22	1.227	0.14	0.100	0.00
	PAB1	22	12.318	0.00	0.110	0.00
	LSM8	22	35.000	0.01	0.650	0.67
	STO1	21	6.905	0.24	0.330	0.03
	SRP1	21	15.810	0.02	0.220	0.00
	ADA2	20	41.650	0.47	0.950	0.00
	SEC22	20	14.050	0.00	0.390	0.06
	PRP19	20	26.350	0.49	0.790	0.37
	NUP42	20	20.650	0.00		0.16
	RPB2	19	21.158	0.37	0.960	0.40
	HSP82	19	2.421	0.00	0.980	0.09
	NUP57	19	31.158	0.01	0.200	0.16
	GAL4	18	31.722	0.00		0.00
	RPG1	18	10.667	0.42	0.720	0.54
	NUP49	18	32.000	0.01	0.210	0.19
	PRP4	18	42.333	0.46	0.730	0.74
	TIF4631	17	5.882	0.14	0.430	0.17
	LSM4	17	41.882	0.39	0.560	0.61
	SIN3	16	2.750	0.41	0.000	0.02
	YKT6	16	11.938	0.08		0.11
	STE11	16	3.875	0.20	0.630	0.00
	LSM7	16	35.313	0.31	0.450	0.53
	PRP6	16	42.000	0.47	0.780	0.78
	SED5	16	10.938	0.00		0.00
	RPO26	16	27.313	0.47	0.620	0.85
	SKP1	16	4.125	0.12	1.000	0.11
	LSM3	16	40.375	0.23	0.560	0.09
	GSP1	16	13.750	0.00		0.00
	PHO85	16	0.563	0.00	0.750	0.00
	SEC17	15	10.733	0.14		0.00
	SMD1	15	27.467	0.45	0.930	0.59
	LSM2	15	37.067	0.32	0.510	0.53
	LSM6	15	40.000	0.34		0.58
	RAD3	15	8.067	0.17	0.590	0.17
	COP1	15	15.600	0.15	0.260	0.04
	NOP58	15	1.000	0.40	0.000	0.00
	NSP1	15	11.533	0.02	0.190	0.18
	BET1	15	14.200	0.00	0.000	0.04
	CBF5	15	4.467	0.31	0.050	0.00
	CKB2	15	9.333	0.23	0.580	0.00
	KEM1	15	19.667	0.06	0.500	0.00
	LSM5	14	38.429	0.32		0.55
	SSA1	14	5.714	0.00	0.340	0.01
	NIP1	14	12.214	0.42	0.650	0.44
	LAS17	14	5.357	0.16	0.390	0.04

Table S1

	HSC82	14	2.357	0.00	0.910	0.09
	SUA7	14	9.429	0.43	0.290	0.04
	CLB2	13	6.538	0.05	0.530	0.00
	CKB1	13	8.462	0.35	0.630	0.00
	SPT16	13	5.692	0.47	0.780	0.00
	RVB2	13	5.231	0.19		0.06
	PRP3	13	39.154	0.43	0.870	0.78
	DCP1	13	18.769	0.00	0.010	0.00
	CDC42	13	3.385	0.00		0.02
	SNF1	13	2.385	0.32	0.640	0.00
	NUP1	13	14.846	0.01	0.080	0.01
	MYO5	13	6.308	0.00	0.390	0.00
	NAM7	13	3.000	0.03	0.240	0.00
	CRM1	13	17.154	0.00	0.030	0.00
	NOP1	13	3.769	0.33		0.00
	RPD3	12	2.750	0.47	0.360	0.03
	SEC23	12	7.583	0.19		0.00
	STE5	12	3.833	0.28	0.480	0.00
	BEM1	12	5.167	0.03	0.030	0.00
	CKA1	12	8.583	0.31	0.580	0.00
	POL1	12	2.333	0.37	0.640	0.00
	RPB8	12	26.500	0.45	0.630	0.90
	SEC31	12	4.000	0.23	0.110	0.02
	RAD51	12	0.917	0.09	0.440	0.00
	TFC4	12	4.667	0.23	0.970	0.27
	CEF1	12	21.083	0.42	0.670	0.44
	BNI1	12	3.250	0.00	0.360	0.03
	ARP4	12	6.333	0.17	0.730	0.05
	ARP2	12	6.667	0.44	0.000	0.05
	SIR4	12	1.583	0.07	0.080	0.00
	PRP43	12	11.583	0.27	0.000	0.41
	CDC53	12	5.000	0.10	1.000	0.12
	KAP104	12	22.000	0.00	0.020	0.00
	CDC25	12	1.917	0.13	0.810	0.00
	CEG1	12	3.667	0.16	0.450	0.00
	NUP145	12	25.083	0.06	0.300	0.45
	SIC1	12	6.417	0.13	0.850	0.01
	TIF4632	12	7.167	0.10	0.420	0.20
	CUS1	12	17.167	0.46	0.690	0.02

mPHs:	mPH	Degree	Motif density	Complex_ratio-same	Localization_ratio-same	Function_ratio-same
	SPT15	40	30.175	0.54		0.25
	TAF10	36	33.750	0.60	0.990	0.24
	TAF14	34	24.118	0.52	0.980	0.44
	PRE1	30	48.867	0.69		0.36
	RPN10	28	50.250	0.70	0.840	0.32
	TAF6	27	47.815	0.54	0.930	0.22
	SRB6	27	51.630	0.82	0.810	0.52
	GLC7	26	6.462	0.94	0.980	0.00
	PRP8	26	38.538	0.50	0.760	0.64
	TAF5	25	46.080	0.60	0.930	0.25
	MRPL9	25	7.360	0.85	0.700	0.85
	RGR1	24	51.125	0.78	0.840	0.58
	SRB5	24	53.458	0.77	0.830	0.52
	MED7	23	48.391	0.87	0.800	0.68
	TAF12	23	51.565	0.59	0.930	0.24
	RPC40	23	23.391	0.82	0.320	0.88
	RPT3	22	44.955	0.63	0.000	0.17
	RPN5	22	49.409	0.74	0.820	0.00
	SRB4	22	51.591	0.82	0.830	0.58

Table S1

MED2	22	47.818	0.89	0.780	0.64
RPT6	22	45.318	0.59	0.800	0.17
TAF9	21	50.190	0.66	0.990	0.27
RPT1	21	48.571	0.66	0.820	0.20
MRPL10	21	8.524	0.76	0.520	0.70
GAL11	21	45.714	0.84	0.830	0.63
RPB5	21	26.571	0.50		0.85
RPN9	20	47.800	0.65	0.810	0.00
PGD1	20	47.200	0.80	0.820	0.64
RPN1	20	49.850	0.60	0.800	0.17
ROX3	20	49.850	0.96	0.780	0.71
MED8	20	47.200	0.86	0.000	0.69
RPN8	19	47.632	0.69	0.820	0.00
RPA135	19	8.000	0.67	0.560	0.75
RPN12	18	50.444	0.68	0.810	0.21
SMD3	18	36.444	0.50		0.73
RPN11	18	44.222	0.66	0.790	0.19
RPO31	18	21.222	0.80	0.500	0.85
RNA14	17	14.235	0.74	0.930	0.33
SPT7	17	32.353	0.66	0.990	0.00
MED4	17	46.353	0.87	0.820	0.69
MSH6	17	4.118	0.80	0.910	0.03
RPN6	17	49.882	0.67	0.800	0.00
TRA1	17	33.706	0.64		0.00
SRB2	17	36.412	0.85	0.780	0.74
SPC24	17	0.471	1.00	0.500	0.50
NGG1	16	36.688	0.62	0.990	0.02
TFP1	16	8.438	0.98	0.700	0.92
SIN4	16	45.688	0.94	0.800	0.72
GCN5	16	35.438	0.64	0.950	0.00
MRPL16	16	8.875	0.77	0.710	0.76
CSE2	16	51.000	0.96	0.820	0.77
PRE6	15	29.267	0.77	0.990	0.77
RPC25	15	26.467	0.80		0.87
RPT5	15	47.533	0.58	0.770	0.19
MED6	15	52.000	0.93	0.810	0.73
SPT8	15	39.267	0.62	0.990	0.02
TAF13	15	31.200	0.81	0.900	0.61
RPN3	15	49.067	0.62	0.790	0.00
PRE2	15	29.067	0.79	1.000	0.75
PRE8	15	26.933	0.71	0.990	0.81
CDC27	14	11.429	0.88	0.730	0.62
SSN3	14	25.357	0.76		0.00
NUT1	14	48.857	0.95	0.790	0.00
POP2	14	3.786	0.75	0.930	0.57
SPP381	14	25.071	0.53	0.830	0.56
RPC34	14	21.000	0.81		0.82
SPT20	14	41.929	0.62	0.990	0.02
RPA190	14	15.929	0.69	0.440	0.88
SPT3	14	41.929	0.62	0.990	0.02
TAF1	14	37.429	0.84	0.960	0.55
SCL1	14	27.071	0.83	0.980	0.65
PAP1	14	13.714	0.96	0.920	0.00
SRB7	14	46.571	0.95	0.800	0.76
CFT1	13	17.308	0.88	0.990	0.37
MYO2	13	2.769	0.68	0.150	0.06
RPC53	13	25.000	0.80	0.500	0.86
TAF2	13	33.077	0.88	0.970	0.67
RPN7	13	49.000	0.70	0.780	0.00
TIF5	13	13.769	0.51	0.710	0.70
REF2	13	14.308	0.98	0.940	0.45

Table S1

	PRT1	13	11.769	0.56	0.690	0.49
	HFI1	13	38.846	0.72	0.990	0.02
	TAF11	13	34.077	0.85		0.68
	CDC23	13	13.154	0.93	0.730	0.67
	PRE10	13	22.846	0.75	1.000	0.82
	CFT2	13	18.692	0.81	0.990	0.34
	RSE1	13	15.308	0.52	0.010	0.00
	APC2	12	13.333	0.99	0.720	0.71
	RPT2	12	51.000	0.63		0.22
	TAF7	12	35.833	0.88	0.970	0.67
	RPT4	12	45.583	0.67	0.770	0.21
	YSH1	12	18.583	0.88	0.990	0.37
	TAF3	12	35.833	0.88	0.970	0.67
	PTA1	12	17.083	0.96	0.990	0.40
	TRS20	12	6.583	1.00	0.250	0.89
	VMA4	12	10.667	0.98	0.730	0.97
	MRPS5	12	2.833	0.94	0.960	0.97
	MSH2	12	3.583	0.64	0.930	0.07

mBH	mBH	Degree	Motif density	Complex_ratio-same	Localization_ratio-same	Function_ratio-same
	SPO12	12				

Note: mBH is the hub without any network motif.
