# Supplementary figures and images for: Hubs with Network Motifs Organize Modularity Dynamically in the Protein-Protein Interaction Network of Yeast
Source: PLoS One. 2007 Nov 21;2(11):e1207. doi: 10.1371/journal.pone.0001207 (PMC2065901; doi:10.1371/journal.pone.0001207)

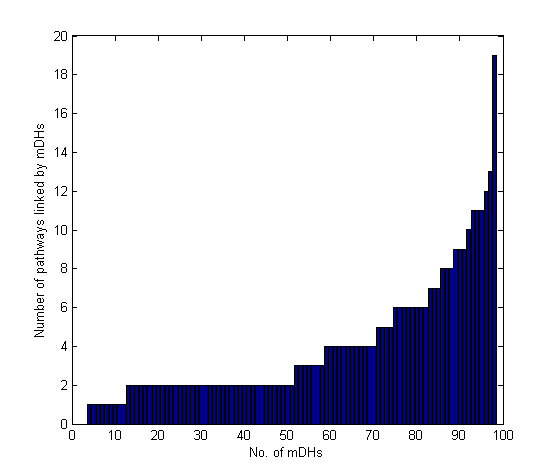

Supplement: Figure S1 — The degrees of mDHs in Figure 5. (0.08 MB TIF) [file pone.0001207.s003.tif]

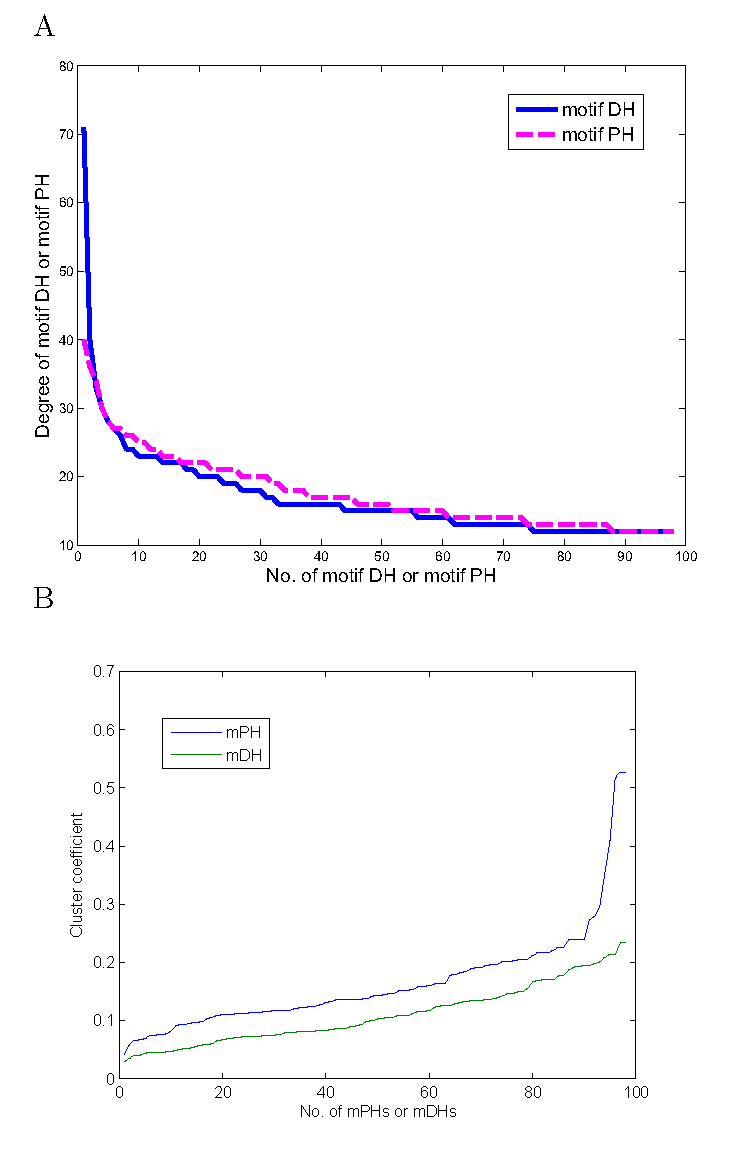

Supplement: Figure S2 — The degree and cluster coefficient differences between mPHs and mDHs. (0.07 MB TIF) [file pone.0001207.s004.tif]

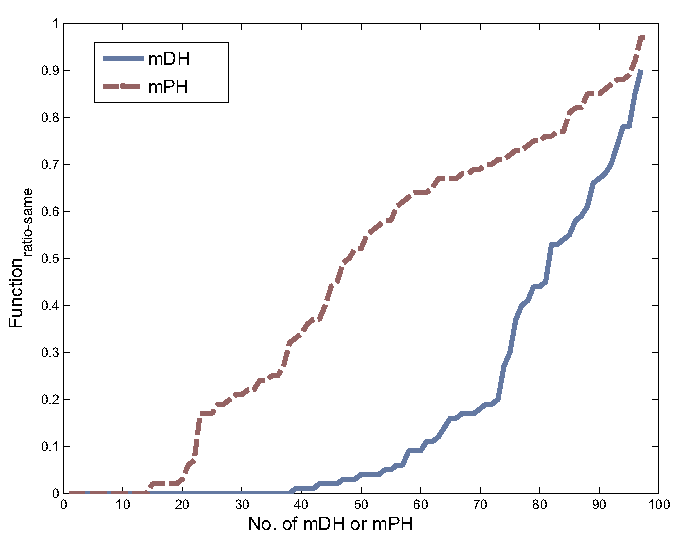

Supplement: Figure S3 — Function_ratio-sames of mDHs is significantly different from those of mPHs (0.04 MB TIF) [file pone.0001207.s005.tif]
